# Atlanta Academy of Medicine

**Published:** 1876-10

**Authors:** R. W. Westmoreland

**Affiliations:** Reporter


### Reports of Societies.

R. W. WESTMORELAND, M.D., Reporter.

Atlanta, July 18, 1876.

Meeting called to order by Vice-President, Dr. J. G. Westmoreland.

Dr. W. F. Westmoreland reported the case of a colored boy, 11 years of age, who received a penetrating wound of the abdomen. One and a half hours after the accident he saw him, and found the intestines protruding from the wound, two or three inches above the pubis. The intestine itself was wounded, but there was no escape of fecal matter—considerable hemorrhage as the result of an artery of the intestine being cut. He determined to close the wound in the intestine with fine silk, by continuous suture, and succeeded in bringing the peritoneal coats in such accurate apposition that the difficulty of healing was considerably lessened. The cut vessel was so compressed by the suture that the hemorrhage was readily controlled. By enlarging the original cut with an incision at right angles, the bowels were returned—silk was used to close the external opening, and the muscles and integument were brought accurately together by deep and superficial sutures.

The next object was to control the inflammation within the bounds most favorable to healing. For this purpose it has been his practice to rely on opium, to the extent of semi-narcotism, for about a week. In the case reported, veratrum was used in conjunction with opium. Up to the present time no bad symptoms have shown themselves. His bowels have not acted during the time he was under the opium, except on one occasion, when, from nausea produced by the veratrum, he had a very slight operation. Is inclined to think the wound in bowel has perfectly healed, or else there would be more disturbance than he has noticed. Stated as his opinion that silver suture is preferable to the silk for deep stiches. In the case reported, on the sixth day discovered that the deep sutures were "cutting," and, on removal, found that in the tract of the suture an immense abcess had formed, which discharged, on being opened, more than an ounce of pus. This, he believes, would not have occurred had the silver been used.

On inquiry, by Dr. Todd, as to what food he was allowed, he replied that he had nothing but water for eight hours after the accident, and then sweet milk.

Dr. Todd inquired what would be his remedy for tympanitis, which would interfere with the sutures by its distension.

Dr. Westmoreland replied that he left the gas to be absorbed—he knew of no remedy that he would use under the circumstances. Remarked that at times the father of the patient noticed at times considerable distension, which would gradually disappear. His object in such cases is to check the peristaltic action of the bowels, and if in doing this the gas accumulates, we can but leave it to be absorbed or pass off in some other manner.

On motion, Academy adjourned.

---

ATLANTA, August 15, 1876.

Dr. H. V. M. Miller in the chair.

Dr. J. T. Johnson reported a case of hemorrhage from the urethra ten days after having performed the operation of internal urethrotomy. At each bleeding the patient lost about half a pint of blood, and what was peculiar, the hemorrhage occurred for three nights in succession at the same hour (about twelve o'clock). There was no hemorrhage during the operation—that is, not more than is usual. The periodicity of the flow suggested quinine, which was given ; no recurrence of it after the third night. The patient made a splendid recovery.

Dr. Elbrey asked Dr. Johnson if he used the catheter or sound on the days preceding the nights the hemorrhages occurred.

Dr. Johnson—On the first day, no ; the second day, yes ; the third day, no.

Dr. Elbrey—Are such occurrences usual with you ?

Dr. Johnson—They were not. Once, upon introducing the sound, had ruptured an artery, which emitted blood profusely for an hour, and the hemorrhage did not cease entirely for three hours.

Dr. Johnson also reported a case of a child with maggots in its eyes, causing considerable inflamation. He carried it to Dr. Calhoun, who extracted nine of the insects.

Dr. Woodhull reported the case of a soldier with stricture, complicated with perineal fistula, occasioned by riding. There were three openings in the perineum. The tissues in the neighborhood of the openings were much swollen, inflamed and tender. Urine came through each tract. The attempt to dilate the urethra with bougies was unsuccessful for months, but his perseverance was finally rewarded by the introduction of the largest size, *per viam naturale.* This treatment was followed by great improvement. The fistulous tracts all gradually healing up, the patient gained twenty pounds of flesh in a short time. The man took syphilis, which caused a relapse, by the position of the chancre.

Dr. Miller asked if he kept a catheter in the bladder.

Dr. Woodhull—No. In two weeks the urine came off by the natural route.

Dr. J. T. Johnson thought he ought to have operated. Said it was exceedingly difficult to cure by dilatation where perineum fistula existed.

Dr. Woodhull also reported a case of constitutional syphilis, of two years duration, in a man aged fifty. He had been under treatment, he said, about that long. He was deaf, eyesight affected, could not go up steps, and the power of co-ordinating muscular movement so much impaired that he frequently fell while attempting to walk. Under twenty-five grain doses of iodide of potass., three times a day, gradually increased to 60 gr. at a dose, the improvement was so rapid that in two weeks he was able to attend to ordinary military duty.

